# Factors associated with good TB infection control practices among primary healthcare workers in the Free State Province, South Africa

**DOI:** 10.1186/s12879-016-1984-2

**Published:** 2016-11-04

**Authors:** Michelle Engelbrecht, André Janse van Rensburg, Gladys Kigozi, HCJ (Dingie) van Rensburg

**Affiliations:** 1 Centre for Health Systems Research & Development, University of the Free State, Nelson Mandela Road, Bloemfontein, 9300 South Africa; 2 Health and Demographic Research Unit, Department of Sociology, Ghent University, Korte Meer 5, Ghent, 9000 Belgium; 3 Department of Political Science, Stellenbosch University, Corner Merriman and Ryneveld Street, Stellenbosch, 7602 South Africa

**Keywords:** TB infection control, Knowledge, Attitudes, Practices, Primary health care, Healthcare workers

## Abstract

**Background:**

Despite the availability of TB infection control guidelines, and good levels of healthcare worker knowledge about infection control, often these measures are not well implemented. This study sought to determine the factors associated with healthcare workers’ good TB infection control practices in primary health care facilities in the Free State Province, South Africa.

**Methods:**

A cross-sectional self-administered survey among nurses (*n =* 202) and facility-based community healthcare workers (*n =* 34) as well as facility observations were undertaken at all 41 primary health care facilities in a selected district of the Free State Province.

**Results:**

The majority of respondents were female (*n =* 200; 87.7 %) and the average age was 44.19 years (standard deviation ±10.82). Good levels of knowledge were recorded, with 42.8 % (*n =* 101) having an average score (i.e. 65–79 %) and 31.8 % (*n =* 75) a good score (i.e. ≥ 80 %). Most respondents (*n =* 189; 80.4 %) had positive attitudes towards TB infection control practices (i.e. ≥ 80 %). While good TB infection control practices were reported by 72.9 % (*n =* 161) of the respondents (i.e. ≥75 %), observations revealed this to not necessarily be the case. For every unit increase in attitudes, good practices increased 1.090 times (CI:1.016–1.169). Respondents with high levels of knowledge (≥80 %) were 4.029 (CI: 1.550–10.469) times more likely to have good practices when compared to respondents with poor levels of knowledge (<65 %). The study did not find TB/HIV-related training to be a predictor of good practices.

**Conclusions:**

Positive attitudes and good levels of knowledge regarding TB infection control were the main factors associated with good infection control practices. Although many respondents reported good infection control practices - which was somewhat countered by the observations - there are areas that require attention, particularly those related to administrative controls and the use of personal protective equipment.

## Background

Healthcare associated infections (HAIs) are responsible for a substantial burden of diseases among patients and healthcare workers, especially in resource-poor settings [1]. The World Health Organization (WHO) reports that at any given time the prevalence of HAIs in developed countries varies between 3.5 and 12 %, and in developing countries between 5.7 and 19.1 % [2]. Of particular concern in high burdened *Mycobacterium tuberculosis* (TB) and human immunodeficiency virus (HIV) settings, is the nosocomial spread of TB among patients and healthcare workers, as a result of poor airborne infection control procedures, and further aggravated by the HIV epidemic as well as the emergence of multi-drug (MDR) and extensively drug resistant (XDR) TB [1, 3–5].

South Africa is classified as a high TB and HIV burdened country, with a TB incidence of 834 cases per 100 000 population [6] and an HIV prevalence among adults aged 15–49 years of 17.9 % [7]. International headlines were made in 2006, with the outbreak of XDR TB in Tugela Ferry, a rural community in South Africa. It was found that in all likelihood the transmission of the XDR TB strain occurred nosocomially [8]. The TB and HIV epidemics are intricately intertwined: approximately 61 % of TB cases are co-infected with HIV and TB mortality among HIV-positive people was estimated to be 134 per 100 000 population [6]. Immune-compromised people are more likely to develop active TB than those with normal immunity levels, i.e. 50–60 % of HIV positive people will develop active disease. The annual risk of TB in an HIV positive person is 10 % compared to a lifetime risk of 10 % in a healthy individual [9].

The dual burden of TB and HIV also severely impacts South African healthcare workers. Occupational exposure to TB is a major health risk for healthcare workers - healthcare workers are three times more likely to acquire TB than the general population [10, 11] and an estimated 81 % of TB cases among healthcare workers are attributable to exposure in healthcare settings [12]. Furthermore, the high prevalence of latent TB Infection (LTBI) among healthcare workers [13] (84 %, 69 % and 62 % using the tuberculin skin test, QuantiFERON-TB Gold-In-Tube and T-SPOT.TB, respectively), especially when compared to the general population, also points to TB being an occupationally acquired disease [14]. The HIV epidemic equally affects healthcare workers, with estimates of the HIV prevalence among healthcare workers ranging from 11.5 to 20.0 % [15].

As TB transmission frequently occurs before an accurate diagnosis is made, it is the responsibility of healthcare workers, particularly managers, to ensure the implementation of appropriate TB infection control measures in all high risk settings so as to ensure that “health care facilities become known as places of healing and safety, not death and contagion” [16]. Good TB infection control relies on the early identification, isolation and rapid initiation of effective treatment of TB suspects combined with good organisation in facilities to avoid overcrowding and ensure appropriate patient flow [10]. More specifically, interventions promoting TB infection control practices in healthcare settings include improving: cough etiquette, respiratory hygiene and natural ventilation in waiting areas and consultation rooms; systematic and regular screening of healthcare workers for TB; and Isoniazid preventative therapy (IPT) and antiretroviral treatment (ART) for HIV positive patients and healthcare workers [1, 10]. South African National TB management guidelines [9], specifically refer to healthcare workers’ increased risk of TB and provide strategies to reduce their exposure to infection: 1) informing healthcare workers about signs and symptoms of TB and encouraging early screening and testing; 2) classifying healthcare workers with signs and symptoms of TB as “high risk TB suspects”; 3) providing voluntary HIV counselling and testing and encouraging healthcare workers to know their HIV status; 4) advocating/providing precautionary measures for HIV positive staff, such as IPT and ART; and 5) appropriate placement of HIV positive staff in low TB risk areas of the facility.

Notwithstanding the availability of guidelines on the implementation of TB infection control practices in healthcare facilities [17, 18], research conducted in South African health facilities found that infection control is often not well implemented [19–27] despite healthcare workers displaying good levels of knowledge about infection control practices [19, 20, 25]. While knowledge may not always be associated with good practices, research has found that motivation and support are priorities for the implementation of interventions to increase healthcare workers’ appropriate execution of TB infection control practices [20].

Very little research currently exists that explores the factors that promote good infection control practices among healthcare workers. This gap is especially pressing at the primary health care (PHC) level, the main point of entry for state-supported TB patient care in many low-and middle-income settings such as South Africa. Healthcare workers often do not have the capacity and resources to correctly interpret, apply and manage policy directives in local contexts. Infection control is dependent on the hierarchy of measures (i.e. facility, administrative and environmental controls as well as personal protective equipment) [17] that together reduce the risk of spreading TB. Research has found that failed facility, administrative and environmental controls means that the focus is on personal protective measure (i.e. the use of N95 respirators), which shifts the burden of managing infection control onto the individual healthcare worker [24]. Accordingly, this study sought to determine the factors associated with healthcare workers’ good TB infection control practices in PHC facilities.

## Methods

### Setting

The Free State, one of the nine provinces in South Africa, is situated in the centre of the country. With a total area of 129 825 square kilometers, the Free State is the country’s third-largest province, with a population of 2 753 200 people. The selected district covers 6 284 square kilometers and is home to 747 431 inhabitants [28]. In 2012, the TB incidence in the Free State was 708.5 cases per 100 000 population, which was higher than the incidence in the country as a whole - 687.3 cases per 100 100 [29]. The HIV prevalence was also higher in the Free State at 19.6 % (15–49 years old) than the national prevalence of 17.9 % [7]. Previous indications are that TB infection control is sub-optimal in some public health facilities in this region [26, 30], underlining the need to determine what influences good infection control practices.

### Design and population

A cross sectional survey as well as facility observations were undertaken at all 41 PHC facilities located in a selected district of the Free State Province, South Africa. Self-administered questionnaires were distributed to all categories of nurses and facility-based community healthcare workers (CHCWs) employed at these facilities.

### Instrument development, data collection and analysis

The survey questionnaire comprising of four sections – demographic information and knowledge, attitudes and practices (KAP) scales related to TB prevention and infection control-was developed based on instruments used in similar studies [20, 21, 25, 31–35]. The knowledge scale contained 22 statements with the response categories “True”, “False” or “I don’t know”. The scale investigated knowledge about: TB symptoms; transmission; personal protective equipment; cough etiquette; ventilation; separation of coughing patients; and TB/HIV. The attitude scale comprised 18 statements with the response categories “Strongly agree”, “Agree”, “Disagree”, or “Strongly disagree”. Topics covered in the scale included: TB/HIV; training; fear of contagion; infection control; personal protective equipment; and occupational health. The practice scale included a series of 12 statements with the response options “Always”, “Often”, “Sometimes” or “Never”. Practices were categorised as relating to the use of personal protective equipment, environmental control activities and administrative control activities.

The knowledge, attitudes and practices scales were formulated to include all levels of TB infection control: *facility-level -* on-site surveillance of TB among healthcare workers as well as IC plans; *administrative control -* early recognition and management of TB; *environmental control -* includes adequate room ventilation; and *personal protection measures – * such as the use of surgical masks and N95 respirators [17]. The questionnaire was translated into the local language, SeSotho, and piloted at two facilities falling outside of the study area. Based on the findings of the pilot study, the questionnaire was revised and adapted. In addition, an observation schedule was developed to assess the actual implementation of TB infection control practices. The schedule was developed based on the WHO levels of TB infection control [17].

Data collection took place between September and November 2015. Researchers visited all 41 facilities and explained the research study to the facility managers, available nurses and facility-based CHCWs. The researchers completed an observation checklist of TB infection control measures. Subsequently the researchers distributed consent forms and the KAP questionnaires in envelopes to all nurses and CHCWs, who were asked to complete the questionnaires in their own time. Arrangements were made for the collection of completed questionnaires on specified dates. Follow-up telephone calls were also made to remind staff that the questionnaires would be collected. Upon collection of the questionnaires, they were edited and respondents were asked to fill out any missing information. While the questionnaires were anonymous, respondents could be traced through the consent forms, which could possibly have encouraged them to report more positive attitudes and better practices than was the case.

The data was captured, cleaned and analysed in IBM SPSS Statistics 23. Data was described using frequency counts and percentages for categorical variables and means and standard deviations for continuous variables. Composite scores were calculated in order to obtain the total knowledge, attitude and practice scores on each of the scales. On the knowledge scale, those respondents scoring <65 % were classified as having a poor level of knowledge, 65–79 % was a moderate level of knowledge and ≥ 80 % was a good level of knowledge. On the attitude scale, respondents scoring 80 % and above were classified as having positive attitudes towards TB prevention and infection control. Respondents scoring ≥75 % on the practice scale were classified as having good practices. The cut-off points for good levels of TB infection control knowledge and practices were informed by a similar study undertaken among healthcare workers in Northwest Ethiopia [36], where those answering ≥60 % of knowledge questions correctly and ≥50 % of practice questions correctly were considered to have good knowledge and practice, respectively.

The outcome variable was good TB prevention and infection control practices, and was defined as all respondents who correctly indicated that they would follow TB prevention and infection control practices ≥75 % of the time. Pearson’s *X* ^2^ test was used to establish any association between independent variables and the outcome variable. Binomial logistic regression analysis was used to determine which factors were significantly associated with good TB prevention and infection control practices. Independent variables included in the model were: sex (male/female), age, attitudes towards TB, whether TB training was received (yes/no), job category (CHCW/assistant or student nurse, professional nurse), location of PHC facility (Sub-districts A, B, C) and knowledge (poor, average, good). The odds ratios (ORs) together with their corresponding 95 % confidence intervals (CIs) were estimated. The significance level considered for this study was 0.05.

### Ethical considerations and authorisation

Ethical clearance was obtained for the study from the Ethics Committee of the Faculty of Health Sciences, University of the Free State (ECUFS No92/2013), and the study was authorised by the Free State Department of Health. All participants completed a consent letter prior to participating in this research.

## Results

### Demographic information

A total of 34 out of 69 CHCWs (49.3 %) and 202 out of 404 nurses (50 %) completed the questionnaires. The majority of respondents were female (*n =* 200; 87.7 %), the average age was 44.19 years (standard deviation ±10.82) and they had been working in their current position for an average of 6.44 years (standard deviation ±8.19). Of the 236 respondents, 129 were professional nurses (54.7 %), 73 assistant/student nurses (30.9 %) and 34 CHCWs (14.4 %). During the 12 months prior to the study, three-quarters of the respondents (*n =* 179; 75.5 %) had received training on TB and HIV-related topics, including infection control, TB/HIV integration, IPT, and intensified case finding. More specifically, 57 % (*n =* 130) had received training in TB infection control measures. Pearson’s *X* ^2^ test was used to establish any association between the demographic variables and TB infection control practices. The only significant association was between sub-district and practices (*p <* 0.05) (Table 1).

**Table 1:** Demographic information

	*n =* 236 (%)	*p*-value
Sex^a^		
Male	28 (12.3)	
Female	200 (87.7)	.374
Sub-district		
A	84 (35.6)	
B	44 (18.6)	
C	108 (45.8)	<.05
Highest formal education		
Secondary/matric	120 (50.8)	
Tertiary	116 (49.2)	.134
Job title		
Professional nurse	129 (54.7)	
Assistant/student nurse	73 (30.9)	
CHW	34 (14.4)	.762
TB/HIV training in the past 12 months^b^		
Yes	179 (77.5)	
No	52 (22.5)	.217

^a^ *n =* 228

^b^ *n =* 231

### Knowledge of TB prevention and infection control

Despite overall good scores on the knowledge scale, 42.8 % (*n =* 101) had an average score and 31.8 % (*n =* 75) had a good score, a closer examination of the individual items revealed the following problematic areas. Less than a quarter of the respondents knew the following correct answers: an HIV positive healthcare worker can still become ill with TB even if they practice infection control (*n =* 49; 20.8 %); TB patients with negative sputum smears are infectious (*n =* 53; 22.5 %); and wearing a surgical mask does not protect healthcare workers from acquiring TB (*n =* 58; 24.6 %). Only half of the respondents correctly knew that having a fever daily for more than a week was a symptom of TB (*n =* 189; 50.1 %) and that an N95 respirator required a fit check (*n =* 119; 50.4 %) – fit testing ensures better protection if the facial seal is correctly fitted [37]. Pearson’s *X* ^2^ test revealed that there was a significant association between TB practices and levels of knowledge (*p <* 0.05) (Table 2).

**Table 2:** Correct knowledge of TB prevention and infection control

Correct responses to knowledge statements	*n* (%)
TB symptoms	
Prolonged cough is a symptom of TB (True)	218 (92.4)
Unintentional weight loss is a symptom of TB (True)	202 (85.6)
Fever every day for more than 1 week is a symptom of TB (True)	189 (50.1)
Night sweats are a symptom of TB (True)	231 (97.9)
TB transmission	
TB patients with negative sputum smears can be considered infectious (False)	53 (22.5)
TB can be spread through blood (False)	210 (89.0)
Patients with TB commonly infect others by talking or singing (False)	112 (47.5 %)
Personal protective equipment	
N95 respirators work just as well when wet or visibly dirty (False)	189 (80.1)
Wearing a surgical mask can help healthcare workers protect themselves from TB (False)	58 (24.6)
An N95 respirator provides an airtight seal on the face that the user does not need to check (False)	119 (50.4)
Cough etiquette	
Patients who are coughing should be given tissues or surgical masks to cover their mouths until TB has been excluded (True)	210 (89.0)
If coughing/sneezing patients or suspects are using tissues or surgical masks, there no need for staff to wear N95 respirators (False)	195 (82.6)
Before a TB suspect has a confirmed diagnosis, having him wear a surgical mask is unnecessary (False)	168 (71.2)
Ventilation	
Mechanical ventilation (like extractor fans) is always more effective than natural ventilation (open windows) for preventing TB (False)	155 (65.7)
Open windows can help prevent the spread of TB (True)	230 (97.5)
If a fan is used in a room, opening windows will not provide additional infection control (False)	191 (80.9)
Separation of coughing patients	
When entering the clinic, every patient should be asked if they are coughing (True)	218 (92.4)
Keeping coughing and non-coughing patients apart in the clinic will help to stop TB from spreading (True)	186 (78.8)
TB suspects in the waiting area should not wait just as long as everyone else, and should not be rushed through the queue (False)	200 (84.7)
TB/HIV	
An HIV positive staff member cancan get sick with TB if they practice TB prevention strategies (False)	49 (20.8)
HIV positive staff who are healthy and on ARVs should still try to avoid working in high risk areas (True)	158 (66.9)
An HIV positive person is more likely than an HIV negative person to become sick with TB if exposed to TB (True)	218 (92.4)

### Attitudes

Most respondent (*n =* 189; 80.4 %) had positive attitudes towards TB infection control practices. Despite these positive attitudes, the majority of respondents were afraid that they would acquire TB at work (*n =* 198; 84.6 %); three in ten reported that their clinic was not concerned about their health and safety (*n =* 73; 31.4 %) and did not provide adequate resources to prevent exposure to TB (*n =* 89; 38.2 %). Most respondents (*n =* 205; 87.2 %) had been screened for TB at work. TB screening for patients entails being asked a series of questions focusing on: cough for more than 2 weeks, weight loss, fever and night sweats. A positive response to one or more of these questions necessitates TB testing. Pearson’s *X* ^2^ test revealed that there was a significant association between TB practices and attitudes (*p <* 0.05).

### Practices

While good TB infection control practices were reported by 72.9 % (*n =* 161) of the respondents (see Table 3), observations (see Table 4) at the facilities revealed this to not necessarily be the case. For example, environmental infection control practices were reportedly well implemented, with 95.2 % (*n =* 216) of the respondents indicating that they opened windows when possible to increase natural ventilation and 91.8 % (*n =* 213) explained to patients why it was important to keep windows open. Observations revealed that 12 (29.3 %) facilities had open windows in all consulting rooms and 9 (22.5 %) facilities had open windows in all waiting areas. This was despite the observation that most facilities (*n =* 35; 85.4 %) had “open window” stickers pasted to their windows to remind them to open windows to prevent TB transmission.

**Table 3:** Self-reported TB infection control practices

	Always	Often	Sometimes	Never
	*n* (%)	*n* (%)	*n* (%)	*n* (%)
Personal Protective Equipment				
I use an N95 respirator when collecting sputum from a patient	106 (52.2)	27 (13.3)	44 (21.7)	26 (12.8)
I use an N95 respirator in the TB consultation room	104 (50.0)	35 (16.8)	37 (17.8)	32 (15.4)
I interact with patients while using a N95 respirator	111 (50.5)	30 (13.6)	49 (22.3)	30 (13.6)
Environmental				
I open windows when possible to increase natural ventilation	216 (95.2)	10 (4.4)		1 (0.4)
I explain to patients why it is important to keep windows open	213 (91.8)	13 (5.6)	5 (2.2)	1 (0.4)
I may turn off fans if they become too noisy or cause cold air to blow around	25 (10.7)	25 (10.7)	55 (23.6)	77 (32.6)
Administrative				
I order sputum specimens when I suspect TB	209 (95.2)	6 (2.7)	7 (3.1)	4 (1.8)
Patients with suspected TB are isolated from other patients	107 (49.1)	24 (11.0)	39 (17.9)	48 (22.0)
I ask each patient who enters the clinic if they are coughing and for how long	205 (88.7)	10 (4.3)	12 (5.2)	4 (1.7)
I move TB suspects to a separate waiting area	113 (53.1)	28 (13.1)	28 (13.1)	44 (20.7)
I move TB suspects to the front of the queue to minimise the amount of time that they spend in the waiting area around other patients	159 (70.9)	27 (12.3)	22 (10.0)	15 (6.8)
I give tissues/surgical masks to coughing patients	145 (67.1)	22 (10.2)	30 (13.9)	19 (8.8)

**Table 4:** TB infection control measures observed at facilities

Observed	*n* (%)
Personal protective equipment	
N95 respirators available	32 (78.0)
TB nurses wearing N95 respirators	5 (12.2)
Environmental controls	
Open window stickers	35 (85.4)
Open window register	12 (30.0)
Open windows in all consulting rooms	12 (29.3)
Open windows in all waiting areas	9 (22.5)
Administrative controls	
Tissues available for coughing patients	7 (17.1)
Face masks available for coughing patients	3 (7.3)
Coughing patients wearing masks	2 (4.9)
Appropriate colour –coded waste bins available in waiting area for tissues/masks	3 (7.3)
Appropriate colour –coded waste bins available in consulting rooms for tissues/masks	37 (92.5)
Separate waiting area for (presumptive) TB patients	11 (26.8)

Poor practices were reported by the respondents when it came to using personal protective equipment: only 52.2 % (*n =* 106) always wore a N95 respirator when collecting sputum from a patient presumed to have TB and 15.4 % never used a N95 respirator in the TB consultation room. Observations at the facilities found supporting evidence for poor personal protective practices, with only five (12.2 %) facilities having TB nurses wearing N95 respirators, despite N95 respirators being available at 32 facilities (78.0 %).

Self-reported administrative controls that were poorly implemented included not separating (presumptive) TB patients (*n =* 100; 46.9 %) from non-coughing patients. This was supported by the observation that only 11 facilities (26.8 %) had separate waiting areas for (presumptive) TB patients. Furthermore, while approximately one- third of the respondents (*n =* 71; 32.9 %) did not always give coughing patients tissues or surgical masks to cover their cough, the reality was that patients at only two clinics were observed to be wearing such masks. In addition, only 7 (17.1 %) clinics had tissues available for coughing patients and only three (7.3 %) had face masks.

### Predictors of good practice

A binomial logistic regression was performed to ascertain the effects of age, TB infection control knowledge and attitudes, TB/HIV training, job category and PHC facility location on good TB infection control practices. Linearity of the continuous variables with respect to the logit of the dependent variable was assessed via the Box-Tidwell procedure. Based on this assessment, all continuous independent variables were found to be linearly related to the logit of the dependent variable. There were six outliers, but no high leverage or influential values. Since the unusual points were not due to data entry errors, it was decided to keep them in the analysis. The logistic regression model was statistically significant, *χ*2 (9) = 29.821, *p <* 0.001. The model explained 19.7 % (Nagelkerke R2) of the variance in the tendency to have good TB infection control practices and correctly classified 74.9 % of cases. Sensitivity was 92.5 % and specificity was 29.8 %, the positive predictive value was 77.1 % and the negative predictive value was 39.3 %. Attitudes and high levels of knowledge were statistically significantly associated with good TB practices after controlling for all other variables in the model. For every unit increase in attitudes, good practices increased 1.090 times (CI:1.016–1.169). Respondents with high levels of knowledge (≥80 %) were 4.029 (CI: 1.550–10.469) times more likely to have good practices when compared to respondents with poor levels of knowledge (<65 %). Although only borderline significant (*p =* 0.05), respondents working in facilities in Sub-district B were 3.024 times more likely to have good TB infection control practices than respondents working in Sub-district A (Table 5).

**Table 5:** Binomial logistic regression analysis of factors associated with good TB prevention and infection control practices

Variables	*n* (%)	Odds Ratio	95 % CI	*P* value
Age (mean and std deviation)	44.9 (10.783)	1.031	0.997–1.067	.078
Attitudes (mean and std deviation)	58.45 (6.045)	1.090	1.016–1.169	.016
Training				
Received no training (ref)	39 (24.8)	1		
Received training	118 (75.2)	0.572	0.232–1.411	.225
Job category				
CHW (ref)	24 (14.9)	1		
Assistant/student nurse	50 (31.1)	0.341	0.102–1.141	.081
Professional nurse	87 (54.0)	0.3725	0.107–1.291	.119
Location of PHC facility				
A	48 (29.8)	1		
B	34 (21.1)	3.024	0.998–9.162	.050
C	79 (49.1)	1.778	0.813–3.887	.150
Knowledge				
Poor (ref)	31 (19.3)	1		
Average	72 (44.7)	1.867	0.822–4.242	.136
Good	58 (36.0)	4.029	1.550–10.469	.004

## Discussion

The increasing evidence of healthcare facility acquired TB in both patients and healthcare workers in South Africa, has led to a greater interest in TB infection control [1, 3, 5, 21, 38]. International and national policies [10, 11] recommend several simple and effective infection control measures aimed at reducing the nosocomial spread of TB in healthcare settings, particularly in resource poor settings. However, as in most endemic settings, healthcare workers in South Africa fail to implement appropriate infection control measures to protect both themselves and their patients from TB infection [19–27, 30]. Our study aligns with these findings, and identified that almost three in ten healthcare workers failed to implement good TB infection control practices in their facilities. As this is based on self-reported compliance, the real situation may be even worse in the facilities. Respondents tend to present themselves in a favourable light (i.e. social desirability bias) [39], and we attempted to counter this by undertaking a number of observations at the facilities, which indeed did reveal poorer practices.

Poor practices relating to administrative TB infection controls were recorded. In particular, and as established in similar research undertaken in South African health facilities [19, 21, 23, 27, 33], we found that approximately half of the respondents always isolated patients with presumptive TB or moved these patients to a separate waiting area. Furthermore, only a quarter of the facilities had separate waiting areas for TB patients. In spite of these poor practices, the majority of respondents knew that separating coughing and non-coughing patients in the facility would help to prevent the spread of TB. A further concern was the inadequate provision of tissues and masks to coughing patients [23, 27], which was supported by observations at the facilities - only two facilities had patients wearing masks on the days of the fieldwork visits. This was the case, despite the majority of respondents reporting that coughing patients should be given masks or tissues to cover their cough. This lack of attention to the implementation of administrative control measures is a serious concern given that these are the first line of defence in healthcare facilities [17] to prevent patient and healthcare worker exposure to TB. Furthermore, these measures are simple and not costly to implement.

Environmental infection control practices were reportedly better implemented than administrative measures and the use of personal protective equipment. The majority of respondents indicated that they opened windows when possible to increase natural ventilation and explained to patients why it was important to keep windows open. However, in practice, and in line with previous research [22, 23, 27, 33, 40], observations at the facilities revealed that only two in ten had all windows open in consultation rooms and waiting areas on the day of fieldwork visits. This was despite the fact that the research was undertaken in the spring, and an even worse picture could present in the winter.

While personal protective measures (i.e. the use of respiratory protection) are the last step in the hierarchy of infection control [17], N95 respirators can filter out TB bacilli and are recommended for use in TB control [41]. Despite these recommendations, poor knowledge and practices were reported when it came to the use of personal protective equipment. This was also found to be the case in other TB infection control studies conducted in South Africa [19, 21, 23, 27, 40, 42]. Moreover, on the days of the fieldwork visits, TB nurses at only five of facilities were wearing N95 respirators.

Overall, respondents had fairly good levels of knowledge about TB infection control, with the majority scoring above 65 % on the scale (c.f. [19, 36, 43]). The main areas of concern related to the infectiousness of patients, use of personal protective equipment, and co-infection with HIV. Attitudes towards infection control were also overwhelmingly positive. In this regard, it was not surprising to find that attitudes and good levels of infection control knowledge were statistically significant predictors of good practices. While other research has also identified knowledge to be a predictor of good practices [36], this was not entirely the case in an Ethiopian study, where TB-related training, and not knowledge, was found to be a predictor of good practices. However, they did find positive correlations between knowledge and practice [43]. To the contrary, our study did not find TB/HIV-related training to be a predictor of good practices. This is supported by Temesgen and Demissie [36] who also found that training on TB infection control did not have an influence on practice. This raises questions regarding the type and quality of training that was provided as it has implications for strategies to improve TB infection control practices. It is possible that the training was too theoretical rather than skills-based. It is important to not only train but also support/mentor healthcare workers on skills to strengthen the implementation of TB infection control strategies [36, 43].

A limitation of the study is the low response rate. The overall response rate of 50 % is low – despite numerous follow up visits to the facilities – but not much different from a similar study [27]. It is well established that healthcare worker participation in self-administered surveys is generally low [27, 44]. Possible explanations for the low response rate include overworked facility staff, large patient numbers and a lack of incentives to complete the questionnaire. Further limitations of the study include self-reports of good practices, although countered to a degree by observations at the facilities, as well as the research taking place in spring, which could also have impacted on practices such as open windows. More robust claims can be facilitated with a longitudinal design, which could also add to the further exploration of causal mechanisms tied to good infection control practices. For example, this study did not investigate the role of patients in ensuring TB infection control in healthcare facilities. Previous research has found that knowledge and acceptability of patient related infection control measures is key to patient adherence not only in healthcare facilities but also in household settings and the community [45].

## Conclusion

Previous research on TB infection control in South Africa mainly focused on hospital settings. The current study addresses this gap by describing practices in PHC facilities (the main entry point for TB patient-care) and exploring factors that promote good infection control practices among healthcare workers. Furthermore, multiple methods were used (self-administered questionnaires and structured observations) to counter the problem of social desirability bias associated with self-reports so as to obtain a more accurate picture of the state of implementation of TB infection control in PHC facilities.

In conclusion, positive attitudes and good levels of knowledge regarding TB infection control were the main factors associated with good infection control practices. Although many respondents reported good infection control practices – which was somewhat countered by the observations – there are areas that require attention, particularly those related to administrative controls and the use of personal protective equipment. Improving infection control practices relies on modifying healthcare worker behaviour. The application of sophisticated behavioural science methods to TB infection control research could lead to better implementation of practices [46]. Conventional training does not appear to improve TB infection control practices, new strategies are required to improve TB infection control in PHC facilities.

## Abbreviations

ART: Antiretroviral treatment
CHCWs: Community healthcare workers
HAIs: Healthcare associated infections
HIV: Human immunodeficiency virus
IPT: Isoniazid preventative therapy
MDR: Multi-drug resistant
ORs: Odds ratios
PHC: Primary health care
TB: Mycobacterium tuberculosis
WHO: World health organization
XDR: Extensively drug resistant
